# The history of sexual selection research provides insights as to why females are still understudied

**DOI:** 10.1038/s41467-022-34770-z

**Published:** 2022-11-15

**Authors:** Malin Ah-King

**Affiliations:** Department of Ethnology, History of Religions and Gender Studies, Stockholm University, SE-106 91 Stockholm, Sweden

**Keywords:** Interdisciplinary studies, Sexual selection, History

## Abstract

While it is widely acknowledged that Darwin’s descriptions of females were gender-biased, gender bias in current sexual selection research is less recognized. An examination of the history of sexual selection research shows prevalent male precedence—that research starts with male-centered investigations or explanations and thereafter includes female-centered equivalents. In comparison, the incidence of female precedence is low. Furthermore, a comparison between the volume of publications focusing on sexual selection in males versus in females shows that the former far outnumber the latter. This bias is not only a historical pattern; sexual selection theory and research are still male-centered—due to conspicuous traits, practical obstacles, and continued gender bias. Even the way sexual selection is commonly defined contributes to this bias. This history provides an illustrative example by which we can learn to recognize biases and identify gaps in knowledge. I conclude with a call for the scientific community to interrogate its own biases and suggest strategies for alleviating biases in this field and beyond.

## Abstract (web summary)

While it is widely acknowledged that Darwin’s descriptions of females were gender-biased, gender bias in modern sexual selection research is less recognized. This Perspective highlights that sexual selection theory and research are still male-centered and suggest strategies for alleviating biases in this field and beyond.

## Introduction

Scientific methodology aims at objectivity, that is for the results of science not to be influenced by subjective factors such as values or personal interests. Yet, history shows that such influences have distorted scientific knowledge; for example, medical science considered female bodies as deviant from a male norm^1^. That is so because scientists are people in a specific time and place whose thinking reflects ‘truths’ as currently accepted by the wider society^2^. Such biases have influenced the questions asked, hypotheses formulated, and interpretations drawn from data—how and why knowledge has been produced^1^. Furthermore, they also impact what we do not know^3^. The history of sexual selection research provides an illustrative example of knowledge/ignorance production by which we can learn to counteract biases.

It is now more than 150 years since Charles Darwin published *The Descent of Man and Selection in Relation to Sex*^4^, and sexual selection has been through an odyssey of disbelief, slumbering existence and blooming, and numerous controversies. In *Darwin and the making of Sexual Selection*^5^, science historian Evelleen Richards convincingly demonstrates how sexual selection history is imbued by its gendered social context. Thus, controversies around females are salient—early on about women’s intelligence, whether female animals have mental capacities for mate choice, and later on about female agency in mating^5^ and fertilization^6^. Darwin’s contemporary colleagues rejected female choice as a mechanism of sexual selection, because they did not think that animals could have the aesthetic sensibility for choice, nor that females were active in sexual encounters^5,7^. When the study of sexual selection was revived in the 1970s, Darwin’s Victorian assumptions about coy and passive females lingered^8,9^. Now, over 50 years later, through a still ongoing process of controversies and negotiations of scientific knowledge, evolutionary biologists have bit by bit moved away from perceptions of females as coy and passive, towards acknowledging that females can have active sexual strategies, be fiercely aggressive, dominant and variable among themselves^6,9^. This history is highly intriguing as it concerns the development of scientific knowledge as well as biases in science; furthermore, it is important for the future of the field. By analyzing this history, we can learn to recognize biases, identify gaps in knowledge and better understand how to counteract such biases in future work.

I use epistemology of ignorance (agnotology)—the examination of how knowledge has been ignored, delayed or not formed^3,10,11^—to explore how knowledge about females has been delayed, or not produced, in sexual selection research. Here, I investigate whether research in sexual selection tends to begin as male-centered and thereafter shift to include female-centered equivalents (a pattern I call male precedence) or vice versa (female precedence). I also investigate how the frequency of publications focusing on sexual selection in males versus in females has changed over time. Thereafter, I discuss the potential causes and consequences of these patterns. Finally, I conclude with suggestions for alleviating biases in this field and beyond, a call for the scientific community to interrogate its own biases and perspectives—in basic assumptions, choice of research questions, study species, methodologies, and interpretations of results, in research priorities by funding agencies, as well as in journals’ assessments in the publication process.

## Areas of male precedence

I searched the literature on the history of sexual selection research, scientific reviews, and papers on the state-of-the-art in and critique of sexual selection to identify male versus female precedence. Even though sexual selection is the outcome of interactions between males and females, I characterize investigations as male-centered (if they focus on the variation in reproductive success among males and thus trait evolution in males) and female-centered (if conversely, they focus on variation in reproductive success among females and trait evolution in females) in order to identify male and female precedence. In my review, the most common pattern was male precedence, whereas female precedence was scarce. Here I illustrate prominent areas of male precedence, followed by contrasting areas of female precedence in the next section.

### Male ornamentation and trait evolution

Most studies of sexual selection have focused on trait evolution in males through variation in male reproductive success, rather than equivalent questions for females. In Malte Andersson’s book *Sexual Selection* from 1994, he provides a review of empirical studies conducted until 1990^12^. Most of those studies (167) concern how male traits evolve through female choice. The majority of these studies investigated sexual selection on male song or display, followed by size and visual ornaments. In contrast, studies on how female traits evolve through male choice comprised thirty studies, mostly on sexual selection for large female size. Of the male-centered studies, the earliest was published in 1944, followed by some studies from the 1960s and an increasing number from the 1970s, whereas the earliest female-centered study was published in 1955, with more studies emerging in the 1970s.

In Andersson’s review^12^, another large segment of studies focused on intrasexual contests, which contribute to sexual selection by influencing variance in mating success. Studies of male contests and related traits such as horns, antlers, tusks, and spurs (58) outnumber, and many also precede, the two studies of female contests (from 1983 and 1988). In addition, the same pattern of male precedence applies to the more specific areas of studies in nuptial coloration in sticklebacks^13^ and birdsong^14^.

### Genital evolution

William Eberhard’s book on *Sexual selection and animal genitalia* (1985) showed that male copulatory organs have been overwhelmingly overrepresented in the study of animal genitalia and introduced the sexual selection framework for studying them^15^. Studying female genitalia has lagged behind, and a review of genital evolution publications in 2014 showed that the field was still male-biased^16^. One recent example of male precedence in genital evolution research is the study of male spermatophores versus the female corpus bursa in butterflies. Butterfly spermatophores and their functions had been studied for many years^12^ before researchers started to investigate the female genital structure that receives spermatophores—the *corpus bursa*—and the function of the toothlike structures inside it, *signa*. Finally, research now investigates the operation of *signa*^17^, the sexually antagonistic coevolution between *signa* and spermatophore envelopes^18^, as well as the enzyme activity of the *corpus bursa*^19^.

### Interpretations of female multiple mating in birds—male infliction or active female choice?

Females were long expected to mate with only one male, especially among socially monogamous birds. With DNA technology, researchers discovered widespread female multiple mating among birds. Behavioral studies of extra-pair copulations indicated that in the majority of species, these were forced^20^. Accordingly, researchers initially interpreted this extra-pair mating as due to males forcing or harassing females to mate multiply^20,21^, or alternatively, that territorial males failed to defend their mates against other males^22^. Thus, early research interpreted extra-pair mating as a male strategy^23–26^. Despite these prevailing interpretations of females as passive, there exist very early accounts of females initiating or readily accepting to mate with other males than their social mate^27^, but these interpretations were ignored or re-interpreted as (possibly) “apparent willingness” rather than “real”^20^. Thus, the prevailing view until the mid-1990s was that male strategies caused extra-pair mating patterns^25^. However, in contrast to prevailing assumptions of passive females, in 1988, ornithologist Susan Smith demonstrated that black-capped chickadee females actively seek out higher-ranked males in their territories to initiate mating^22^. Since then, there has been a slow shift towards acknowledging female multiple mating as an active female strategy, at least in some species^6^.

### Sperm competition versus cryptic female choice

**Fig. 1:**
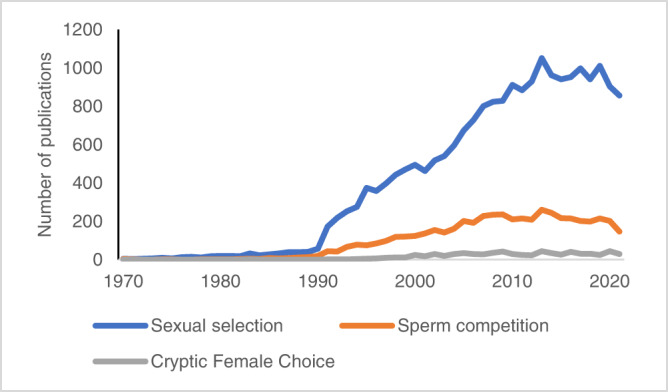
Publications on sperm competition versus cryptic female choice. Comparison of number of publications on sexual selection (blue line), sperm competition (orange line), and cryptic female choice (gray line) over time. Sperm competition studies by far outnumber cryptic female choice studies. The total number of papers published on sperm competition is 5219 compared to 728 on cryptic female choice, and the ratio for year 2021 is 147 versus 29. The number of publications were retrieved from Web of Science searches in “all fields” for “sexual selection”, “sperm competition” and “cryptic female choice” (June 6, 2022).

Darwin’s sexual selection theory concerned competition that occurred before mating. In 1970, Geoff Parker’s theory of sperm competition extended sexual selection theory to events occurring after copulation, with its male-centered focus on competition between sperm from different males for the fertilization of a set of eggs^28^. The equivalent female-centered theory, cryptic female choice (the female influencing which sperm fertilize her eggs) was developed by Randy Thornhill and colleagues some years later^29–31^. The sperm competition field developed before that of cryptic female choice, and is still many times more productive in terms of published papers (Fig. 1).

### Infanticide

Primatologist Sarah Blaffer Hrdy was the first to suggest that infanticide by males of unrelated infants could be a sexually selected strategy in 1974^32^. She reasoned that if a male killed a female’s unweaned infant, the female could conceive his offspring quicker and thereby increase his reproductive success. Although both males and females may commit infanticide of unrelated infants, among the multiple hypotheses suggested, it is only when males are perpetrators that the sexual selection hypothesis is suggested as an explanation, because males potentially increase their reproductive success by reducing female remating latency^33^. For females, on the other hand, committing infanticide produces no equivalent effect, but may increase their reproductive success through, for example, resource competition, which is then explained by natural selection. Therefore, sexual selection research includes a multitude of studies on males committing infanticide^34^. Reviewing the sexual selection and infanticide literature (Web of Science search of “all fields” for “infanticide and ‘sexual selection’” May 27, 2022), the majority of studies (109 of 122) describe sexually selected infanticide as a strategy restricted to males. However, since 1991, some studies (13), especially on birds, describe it as a sexually selected strategy in both sexes (both sexes may increase their fitness by killing unrelated offspring, however, females compete for opportunities to reproduce rather than to mate), and the subject of sexually selected infanticide by female animals has gained more attention^35–37^. Thus, there is male precedence in the literature on sexually selected infanticide, but as several authors have pointed out, whether or not infanticide by females is considered sexual selection depends on how sexual selection is defined, which I discuss further below.

### Sexual conflict

Although many issues in sexual selection involve conflict between the sexes (sexual conflict), such as infanticide, the sexual conflict field only emerged in the 1990s and has focused particularly on male reproductive traits that impose a cost on female fitness during or after copulation^38,39^. In their 2011 investigation of the field, Kristina Karlsson Green and Josefin Madjidian point out that theoretical models of sexual conflict primarily focus on traits which incur benefits to males and costs for females^40^. Moreover, Melissa Plakke and colleagues assert that equivalent research on females has lagged behind, but that in all systems where females have been meticulously studied, female reproductive adaptations of sexual conflict have been identified^19^. Thus, there was male precedence in the sexual conflict field.

Furthermore, Marlene Zuk and colleagues assert that early studies of sexual conflict were biased towards a few insect model species which exhibit more or less intense sexual conflict, and in which sexual conflict is costly to females. As the model systems in sexual conflict studies diversified over time, the support for the view that sexual conflict always results in harm of females decreased^41^. Thus, the choice of model species influences the conclusions drawn.

## Areas of female precedence

While there are more areas with male precedence, a few areas show female precedence. Models about the evolution of mate preferences have primarily focused on female preferences^12^. For example, the idea that female sensory biases (preferences that have evolved in a non-mating context and which are not necessarily sex-specific) make them prefer certain male traits in mate choice, preceded the suggestion that males may also have sensory biases for female traits^42,43^.

Another possible example of female precedence is maternal and paternal effects, that is when a parent affects the phenotype of offspring through other means than genetic inheritance. A review on maternal and paternal effects and sexual selection gives examples of females that mate with attractive partners providing more resources to the offspring (maternal effect studies from 1986, 1998, 2000)^44^. However, among their empirical examples of parental effects on sexually selected traits, there is no clear pattern of female precedence.

Yet another potential example of female precedence is priming, that a hormonal stimulus may influence reproductive condition and behavior. For example in rodents, male odor can induce maturation and ovulation in females (which might be a sexually selected adaptation)^45^.

## Quantifying studies on sexual selection in males versus females

**Fig. 2:**
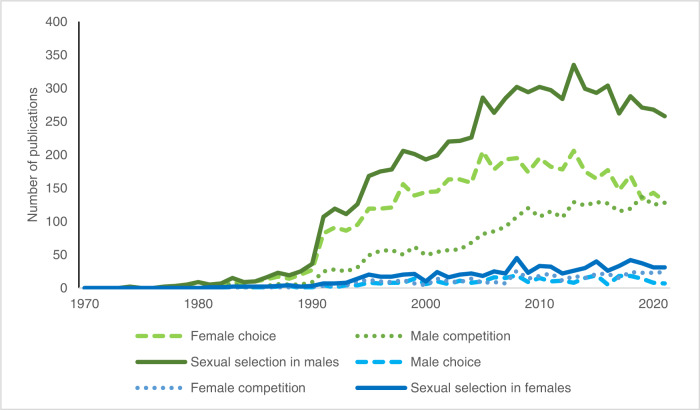
Publications on sexual selection in males versus females. Number of publications on female choice (green dashed line) and male competition (green dotted line), both mechanisms concern variance in male reproductive success, and added together gives the number of publications on sexual selection in males (green solid line)—compared with publications on male choice (blue dashed line) and female competition (blue dotted line), mechanisms which concern the variance in female reproductive success, which added together gives the number of publications on selection in females (blue solid line). The figure shows that studies investigating sexual selection in males outnumber studies of sexual selection in females. Data from Web of Science (June 6, 2022).

I compared the volume of publications focusing on sexual selection in males versus those focusing on sexual selection in females. Specifically, I compared the numbers of studies on variance in male reproductive success (identified by the search terms “female choice” and “male competition”) to those on variance in female reproductive success (“male choice” and “female competition”). Although this is a rough measure, it shows that studies in the field are more frequently focusing on sexual selection in males than on sexual selection in females (Fig. 2), which suggests that sexual selection in females is understudied. Studies of both male choice and female competition are few throughout the period, and very few in comparison to studies on female choice and male competition.

## Causes and consequences of bias

Although it may be difficult to attribute a biased research pattern to a specific kind of researcher bias, it is important to recognize that there are different kinds of biases. Secondary sexual characteristics are usually more developed in males, especially the extravagant plumages in birds. Such extraordinary ornaments posed a challenge to Darwin’s theory of evolution by natural selection as they should decrease survival, and thus it is not surprising that bird ornaments and song were central to Darwin’s ideas about sexual selection ^5^. Similarly, it seems reasonable that contemporary research started out with the more visible male characteristics and that studies of the less conspicuous equivalent female traits followed later, though in a few species these traits are even more pronounced in females than in males^46^. Furthermore, in some cases there may be practical reasons that females are understudied. For example, it can be more difficult to study internal, often soft tissue female genitalia and the process of gamete selection within the female body (or methods for doing so have not been established yet), compared to studying external male genitalia and testing some predictions of sperm competition (e.g., counting sperm and weighing testicles).

Yet, not all the bias can be explained by the biological features of the study systems. Presumptions about sexual selection being weaker or non-existent in females, as well as assumptions of females as “coy” and passive, have led to an over-emphasis on sexual selection in males and disregard of evolution in females^2,8,9^. For example, there is no logical reason that the female-focused idea (cryptic female choice) should emerge later than the male-focused one (sperm competition) when sexual selection theory was expanded to include events after mating. This may instead reflect gender bias by androcentrism (male centeredness, which is primarily focusing on features associated with males and failing to study features associated with females^47^.

Still another cause of bias is the currently prevailing definition of sexual selection, which excludes many ways in which females compete for reproduction.

### A bias due to the definition of sexual selection itself

**Fig. 3:**
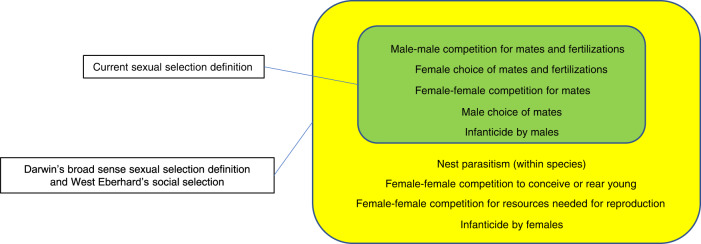
Inclusion/exclusion of selection mechanisms depending on definition. Illustration of which selection mechanisms are included in the currently prevailing definition of sexual selection compared to Darwin’s broad sense sexual selection and West Eberhard’s social selection.

Darwin provided two definitions of sexual selection: in *Origin of Species* (1859)^48^, his first definition was “Sexual selection… depends, not on a struggle for existence, but on a struggle between the males for possession of the females; the result is not death to the unsuccessful competitor, but few or no offspring”^48^. This is a narrow sense definition^49^, since it is restricted to sexual selection in males and only includes competition for mates. Later, in *the Descent of Man and Selection in Relation to Sex*, Darwin wrote “Sexual selection … depends on the advantage which certain individuals have over other individuals of the same sex and species, in exclusive relation to reproduction”^4^. This broad-sense definition is more inclusive because it encompasses sexual selection in both sexes as well as competition beyond the struggle for mates. Even more than 150 years later, there is an ongoing debate about how to define sexual selection^50^. In practice, the currently most prevailing definition of sexual selection is “differences in reproduction that arise from variation among individuals in traits that affect success in competition over mates and fertilizations”^12,51,52^. Though this includes both females and males, it is a narrow-sense definition because it excludes many ways in which females compete among each other for reproduction—such as competition for opportunities to breed or rear young^53^, nest parasitism (laying eggs in other females’ nests), female–female aggression (for resources other than mates)^49,54^, female competition for offspring care^55^ and infanticide by females^35^. How to solve this problem is up to the research community, but is important to be aware that the prevailing terminology largely excludes female competition for reproduction and thereby produces ignorance about broad-sense sexual selection in females (Fig. 3).

### Consequences of bias

**Fig. 4:**
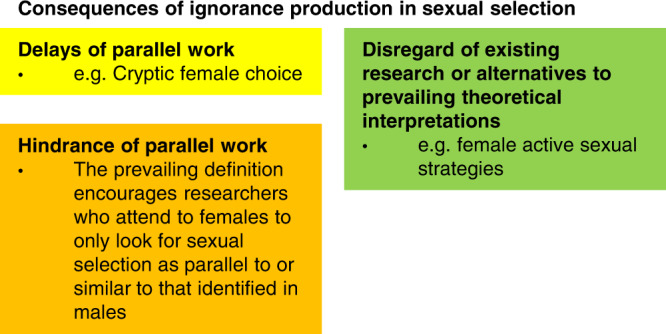
Consequences of ignorance production in sexual selection. Although the higher frequency of extraordinary male ornaments in males may partly explain the prevalent male precedence (that is research starts with focusing on male-centered investigations or explanations and thereafter include female-centered equivalents), it is also partly due to gender bias. Such gender bias has delayed or hindered parallel work on females as well as led to disregard of existing research and alternative theoretical interpretations.

Male precedence is a kind of androcentrism. Although the prevalent male precedence in sexual selection research can partly be attributed to biological patterns, partly it is also due to cultural gender bias. The history of sexual selection research shows that overfocusing on males has obscured the role of females as well as the evolution of females^56^. Ignorance has been produced through delays or hindrances of knowledge production about female evolution^2,9,19^, and disregard of existing research or alternative interpretations^6,9^ (Fig. 4). One example of such gender bias was demonstrated in Marcy Lawton and colleagues’ analysis of ornithological research^57^. In a book on pinyon jays, the authors dismissed female aggression as the bird version of PMS, rather than recognizing that females hold territories and rule dominance hierarchies in this particular species.

Shifting away from androcentric assumptions in the field has repeatedly expanded our understanding of sexual selection—about female ornamentation^13^, female birdsong^14^, the role of females in extra-pair copulations^58^, and fertilization^59^. Likewise, shifting from stereotypical assumptions about males has led to new insights in male choice and sperm allocation^58^. Challenging of androcentric assumptions about passive females led to the recognition that females have active sexual strategies, informed new theory^60–62^, and resulted in a more comprehensive understanding of sexual selection^47,58,63^. However, the prevailing definition of sexual selection still encourages researchers who attend to females to only look for sexual selection as parallel to or similar to that identified in males.

### Suggested solutions for the inclusion of female competition

One proposed solution has been to use Darwin’s broad-sense definition^46^, whereby female competition would be categorized as sexual selection, corresponding with male competition. However, Darwin’s broad-sense definition, in particular, brings other problems with distinguishing between sexual and natural selection^50^. Furthermore, a review on female ornamental traits by Joseph Tobias and colleagues shows that within the same species, the same trait often has different functions for females and males^64^. For example, reindeer antlers are sexually selected in males, whereas females use them in competition for access to foraging sites. Furthermore, for both female and male ornaments there is a continuum of functions between sexual selection and non-sexual social competition, although ecological competition is more common in females than in males^64^. Sexual selection is often assumed to be the cause of female ornamentation even though it may have other anti-predator or ecological competition functions. Likewise, sexual selection as an explanation for male ornaments may be overestimated, because males also compete socially for other resources than mates and fertilizations. Thus, using Darwin’s broad sense definition does not resolve the problem that similar traits can be the result of different forms of selection^64^.

A suggested alternative solution is to use Mary Jane West Eberhard’s theoretical framework of social selection (a sub-type of natural selection, including the sub-type sexual selection)—defined as “differential reproductive success (ultimately, differential gene replication) due to differential success in social competition, whatever the resource at stake”^65^. This theoretical framework includes the many different ways in which social competition and cooperation occurs in females and males^49,64,66^ (Fig. 3). The narrow sense definition of sexual selection has shortcomings for both sexes, but it works better for males, whereas the framework of social selection is particularly relevant for females^64^. Social selection is a layer of selection that is so far underappreciated^49,64,66^.

## Suggestions for alleviating biases in sexual selection and beyond

Here, I provide suggestions for alleviating biases (see also Box 1). As scientists, we all have our own biases—our theoretical frameworks^9,16,57^, our human senses (which enable certain insights into animal behavior but hinder others^67^, study species^16,41^, geographical locations^14^, cultures^68^, and experiences^8,14^. Accordingly, science historians and philosophers have shown that the exclusion of certain people based on sex and race has influenced the science produced^69^. Recently, Casey Haines and colleagues showed a connection between an increase in women authors investigating female birdsong and a paradigm shift in this area—from assuming birdsong to be a sexually selected male trait to concluding that female birdsong is widespread and that singing by both sexes is ancestral in birds^70^. The same pattern was noted in the history of primatology, in which a number of pioneering women primatologists with feminist perspectives started to study females in their own right—thereby developing new research questions, challenging language use, and producing new theoretical understandings—leading to a general shift in the field^63,71^. In addition, Japanese primatologists interested in a holistic understanding of primate societies, early on recognized female dominance hierarchies that Western primatologists overlooked^68^. In line with these developments, science philosopher Helen Longino argues that research constructed by an adequately diverse community with an open critical dialog and serious consideration of all relevant perspectives are important for enabling progress in scientific knowledge^72^.

Which fractions of nature become known and which remain unknown is the result of negotiations in society and the scientific community^1^. The inclusion of critical analyses of gender bias can improve science^1,57,72,73^, and healthy working relationships between those performing gender analyses of science and those doing science can speed integration of critical analysis of gender bias in science^1^. There is always a multiplicity of theories in a scientific community, and ideological influence, such as androcentric bias, is probably strongest when it leads to the ignorance of certain theoretical interpretations^73^. Therefore, one way of developing science is to explore marginal theories that have been left out because they are perceived as being outside the prevailing paradigm^73^. One way to boost new research is to gather expertize on understudied perspectives in a specific area, as was recently done in a workshop on female reproductive biology^74,75^.

When designing experiments, these reflective questions can be useful: Are the assumptions, theoretical background presentation, terminology, and interpretations liable to gender stereotypical bias?^76^ Are gender stereotypes perpetuated by ascribing emotional states to behavior (e.g., aggressive, rapacious, voracious)? In sexual selection experiments, is sexual/social selection in both females and males taken into account?

Being aware of biases enables constructing experimental controls^77^, therefore it might be useful to ask colleagues that are least likely to conform to the investigator’s way of thinking to review the experimental design. It may not be possible to test all alternatives in practice, but it is important to always be aware of the broader theoretical possibilities, and consider if the experiment can rule out potential alternative explanations.

When choosing which species to work with, consider what Zuk and colleagues showed, namely that conclusions drawn are influenced by the model system used^41^. Diversifying model systems gives broader perspectives on research questions. Moreover, useful methods for eliminating observer bias are (when possible) “blind” observations, done by investigators who are not aware of the hypothesis being tested, and analyzing subsets of data independently to ensure inter-observer reliability of coding behavior^78^. Furthermore, publishing null results and making replication studies is important to counteract publication bias^79^.

Changing scientific priorities are enabled by funding opportunities, institutional arrangements, and by the scientific community—therefore funding agencies, institutions, journals, editors and reviewers all influence which proportions of nature become known and which are neglected^1^.

Box 1 General strategies for counteracting bias in sexual selection research and beyond It is important to acknowledge biases and the role of diversity in the scientific community for contributing to previously understudied issues in science. Here follows a list of suggestions to counteract bias at different stages in the research process. Importantly, funding agencies, institutions, journals, editors, and reviewers all influence the research community’s priorities of which proportions of nature to become known and which become neglected^1^.

- In planning research: be wary of stereotypical assumptions and that definitions are important for the inclusion/exclusion of issues in an area; recognize that the study subjects enable certain investigations and preclude others; consider alternative theoretical models and hypotheses.
- In conducting research: consider potential biases to design experimental controls; use “blind” observations (done by investigators who are not aware of the expected experimental outcome) when possible, and independent analyses of coding behavior to ensure inter-observer reliability.
- Interpreting results: be aware that language use and terminology shape interpretations, for example, ascribing emotional states to behavior (e.g., aggressive, rapacious, voracious) can perpetuate stereotypes and lead to confirmation biases. Gender-neutral language and specific operational terminology are useful for reducing gender bias.
- For research evaluators and publishers: be wary of unconscious bias; recognize the value of open critical dialog and serious consideration of all relevant perspectives; counteract publication bias by publishing null results and replication studies.

## Summary

The history of sexual selection research shows prevalent male precedence which has, in particular, delayed knowledge not only about sexual selection in females, but also more broadly about reproduction and evolution in both females and males. This prevalent male precedence can partly be explained by the biological features that are more obvious and more amenable to study, but is also partly due to cultural gender bias. History also shows repeatedly that abandoning stereotypic assumptions about passive females has led to progress in the field.

The quantification of publications in sexual selection shows that studies have historically and continue to focus much more on sexual selection in males than in females. This result in part reflects that sexual selection is often stronger in males, but also that a lot of what females do in terms of competition for reproduction is not included in the prevailing narrow-sense definition of sexual selection—such as female-female reproductive competition for resources other than mates, breeding opportunities to conceive or rear young and laying eggs in other females’ nests. The focus on narrow-sense sexual selection has perpetuated ignorance production about the selection on females. Thus, sexual selection research is still heavily focused on sexual selection in males while sexual selection in females is understudied. Awareness of this bias and increased efforts to counteract it would result in a more comprehensive understanding of evolution. The history of sexual selection illuminates how we can learn to recognize biases and identify knowledge gaps, suggesting strategies for alleviating biases in this field and beyond (Box 1).
